# Ground reaction forces during walking with different load and slope combinations in rats

**DOI:** 10.1186/s40634-017-0102-8

**Published:** 2017-08-31

**Authors:** N. Bravenboer, B. T. T. M. van Rens, H. W. van Essen, J. H. van Dieën, P. Lips

**Affiliations:** 1 Amsterdam Movement Sciences, Department of Clinical Chemistry, VU University Medical Center, PO Box 7057, 1007 MB Amsterdam, the Netherlands; 2 Amsterdam Movement Sciences, Faculty of Behavioural and Movement Sciences, Vrije Universiteit Amsterdam, Amsterdam, the Netherlands; 3 Department Internal Medicine, VU University Medical Center, Amsterdam, the Netherlands

**Keywords:** Animal model, Exercise, Loading, Osteoporosis, Bone

## Abstract

**Background:**

Treadmill animal models are commonly used to study effects of exercise on bone. Since mechanical loading induces bone strain, resulting in bone formation, exercise that induces higher strains is likely to cause more bone formation. Our aim was to investigate the effect of slope and additional load on limb bone strain.

**Methods:**

Horizontal and vertical ground reaction forces on left fore-limb (FL) and hind-limb (HL) of twenty 23-week old female Wistar rats (weight 279 ± 26 g) were measured for six combinations of SLOPE (−10°, 0°, +10°) and LOAD (0 to 23% of body mass). Peak force (Fmax), rate of force rise (RC), stance time (Tstance) and impulse (Fint) on FLs and HLs were analyzed.

**Results:**

For the FL, peak ground reaction forces and rate of force rise were highest when walking downward −10° with load (Fmax = 2.09±0.05 N, FLRC = 34±2 N/s) For the HL, ground reaction forces and rate of force rise were highest when walking upward +10°, without load (Fmax = 2.20±0.05 N, HLRC = 34±1 N/s). Load increased stance time. Without additional load, estimates for the highest FL loading (slope is −10°) were larger than for the highest HL loading (slope is +10°) relative to level walking.

**Conclusions:**

Thus, walking downward has a higher impact on FL bones, while walking upward is a more optimal HL exercise. Additional load may have a small effect on FL loading.

**Electronic supplementary material:**

The online version of this article (doi:10.1186/s40634-017-0102-8) contains supplementary material, which is available to authorized users.

## Background

The physiological mechanisms that underlie bone mechano-responsiveness are usually studied in animal models, especially in rats, for which different types of non-invasive exercise interventions have been developed. These interventions include voluntary wheel (Aikawa et al. 2015; Fonseca et al. 2011), treadmill running (Bennell et al. 2002; Chen et al. 1994; Chen et al. 2011; Clarke 1995), treadmill running with additional load (Tromp et al. 2006; Bravenboer et al. 2001; Van der Wiel et al. 1995), climbing (Mori et al. 2003), jumping (Honda et al. 2003), and weight lifting (Wirth et al. 2003). Of these interventions, those that involve walking or running require less training time to familiarize the rats with the task. Furthermore, applying additional load, consisting of weight in a backpack is a simple but effective method to increase the mechanical stimulus. This type of training could even be extrapolated to humans, in whom it might be beneficial for prevention of bone loss in the elderly.

The effects of running exercise on bone mass have so far been equivocal. Several studies have reported that low velocity treadmill running stimulates bone formation only in growing animals (Hamann et al. 2012; Ju et al. 2012; Huang et al. 2003). Conversely, Bennell et al. (2002), who compared 5 weeks old female rats with 17 weeks old female rats, concluded that age does not influence the bone response to treadmill exercise. Nevertheless, when rats were running with an additional load, bone mineral density increased more compared to running without additional load (Van der Wiel et al. 1995). In addition, climbing, a form of high resistance exercise that likely amplifies the mechanical stimulus, more noticeably increased bone mass than running on the treadmill (Jung et al. 2014). These results indicate that bones which experience the largest strain relative to habitual loading might have the largest potency to show a response to mechanical loading (Warner et al. 2006). In addition, the osteogenic response can be influenced with slope adjustments of the treadmill. Reports that compare uphill running and downhill running are scarce. Though, uphill running was compared to swimming in mice, showing uphill running had less pronounced effects on bone mass than swimming (Warner et al. 2006), downhill running in contrast seems to be a potent osteogenic stimulus in the femoral metaphysis (Hamann et al. 2012).

Mechanical load-induced osteogenic response occurs through deformation – strain – of loaded bone in a dose responsive manner. Additional load, which apply higher forces to a bone, is therefore likely to induce a higher strain. In limb bones, bone strain showed to be proportional to the magnitude of the ground reaction forces during gait under varying conditions in several species (Rubin and Lanyon 1985; Biewener 1991; Main and Biewener 2004). For this reason, ground reaction force could be used to measure bone strain. Since the association between ground reaction forces and strain is general among species, extrapolation towards humans might be feasible. This, in combination with the non-invasive application of additional load, may result in an effective therapy to prevent bone loss in osteoporotic elderly.

We aimed to study the effects of additional load on ground reaction forces of rat fore limbs (FL) and hind limbs (HL). Since additional load results in an osteogenic response, we assumed additional load will increase the ground reaction force. Secondly, we aimed to study the effects of different slopes on ground reaction forces of rat FLs as well as HLs. Since downhill walking or running costs less energy in both rats (Armstrong et al. 1983) and humans (Margaria et al. 1963), it may be a more feasible exercise regimen in both rats and humans.

## Methods

This experiment was conducted at the VU University Center for Experimental Animal Research (Amsterdam, the Netherlands), in accordance with the Dutch law on the protection of animals and was approved by the Institutional Animal Care and Use Committee of the ‘Vrije Universiteit Amsterdam’.

Thirty female Wistar rats (Harlan, Horst, the Netherlands) were obtained immediately after weaning, at the age of age 22 days. The rats were socially housed, 3 per cage, in standard type 4 macrolon cages, with ad libitum available water and food (Teklad global 16% protein rodent diet, Harlan, Horst, the Netherlands). The rats were kept in a controlled environment of a fixed 12:12 light-dark cycle with room temperature and daily moisture maintained at 21.2±0.3° and 55.4±5.8%, respectively. From the age of 22 days to 35 days body mass was determined five times per week and twice weekly after the age of 35 days.

**Fig. 1:**
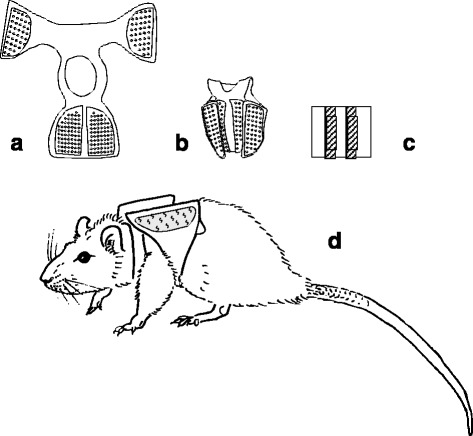
Placement of the additional load in the backpack. **a** Placement of the Velcro, used for closing at the dorsal side, on the chamois leather backpack. **b** dorsal view of a closed backpack. **c** Load, prepared from leaden strips is symmetrically piled (1 cm from each other) on a piece of Velcro, which can be attached to the corresponding velcro on the backpack, thus closing the backpack tightly. **d** Rat carrying a backpack without load. The additional load was attached to corresponding velcro on the backpack, therefore, the leaden strips were always located bilateral from the spine, starting above the armpit

A random selection of 20 rats was trained to walk along the length of a one meter long tunnel with a built-in force plate, with additional load in a backpack (Fig. 1; Bravenboer et al. 2001). At the age of 23 weeks, ground reaction forces of their left FLs and HLs were measured with and without load and for three different slopes: −10°, 0°, +10°.

The rats were trained to walk through the tunnel for 20–30 min per day, five days per week, for 19 weeks. The load in the backpack gradually increased from 10 to 23% of body mass at the age of 17 weeks. Previous experiments demonstrated that rats were able to carry this maximum load in the backpack (Bravenboer et al. 2001; Tromp et al. 2006).

The tunnel contained a KAPAplast force plate inlay (Fujifilm Sericol Nederland BV, Lochem, the Netherlands) that was supported by two horizontally and two vertically oriented 4.5 N load cells (L2357 S-Beam junior load cells, Futek, Irvine, USA). Due to their insignificance (Muir and Whishaw 1999), the mediolateral components of ground reaction forces were not measured. The width between the tunnel’s Perspex walls could be adjusted to the size and walking pattern of the rat, guiding the rat to walk through the middle of the path, with only its left FL and HL on the force plate. The gait kinetics’ symmetry allowed for left limbs only examination (Muir and Whishaw 1999). Trials in which bipedal contact (or partial contact of one limb) with the force plate occurred were excluded from the analysis. The slope of the walking tunnel changed from −10° to +10° in 5° steps. The walkway, including the force plate, was covered with anti-slip coating (Alabastine, Ammerzoden, the Netherlands). The rat trainer measured five rats per day in a random order, first with the additional load and subsequently without. Data were collected when the rat crossed the force plate. Each run’s output of the load cells was amplified and converted from analog to digital (Porti, TMS International BV, the Netherlands), at 1000 samples per second and 22-bits resolution, and stored on hard disk. Each trial was filmed with a high speed camera to check the force-plate measurement reliability. At least 4 successful measurements were used for further calculation.

### Data processing

Two thousand ninety-three of 3808 measurements, verified by video recording, were processed using Matlab (The Math Works Inc., Natick, MI). Vibrations of the force plate were filtered out with use of a Butterworth filter (order 2, cut-off frequency 30 Hz). Measurements with a time-force plot, in which contact of at least one FL and one HL, were clearly visible and distinguishable (*n* = 1721) were selected for further analysis. Per limb, start and end point, and peak maximum, representing peak force, were automatically determined in the resultant forces from the Fhor and Fvert vectors (Fig. 3). Overlapping ends and beginnings of consecutive peaks were extrapolated linearly, using the regression coefficient of the samples between the point of intersection and the value that represented 20% of the peak force. If correct, start and end point, maximum reaction force, area under the force-time peak, and the rising slope regression coefficient were determined.

### Statistics

Data were analyzed with SAS (SAS Inst., Cary, NC). Multiple comparisons were performed with Tukey-Kramer adjustments. The effects of SLOPE (−10°, 0°, +10°) and LOAD (yes = 1, no = 0) were examined for FL and HL separately using a mixed model which included rat number (RAT) as a random variable and SLOPE, LOAD and their interaction as fixed variables. If the interaction SLOPE*LOAD was not significant (i.e., *P* > 0.01), it was excluded from the model.

**Fig. 2:**
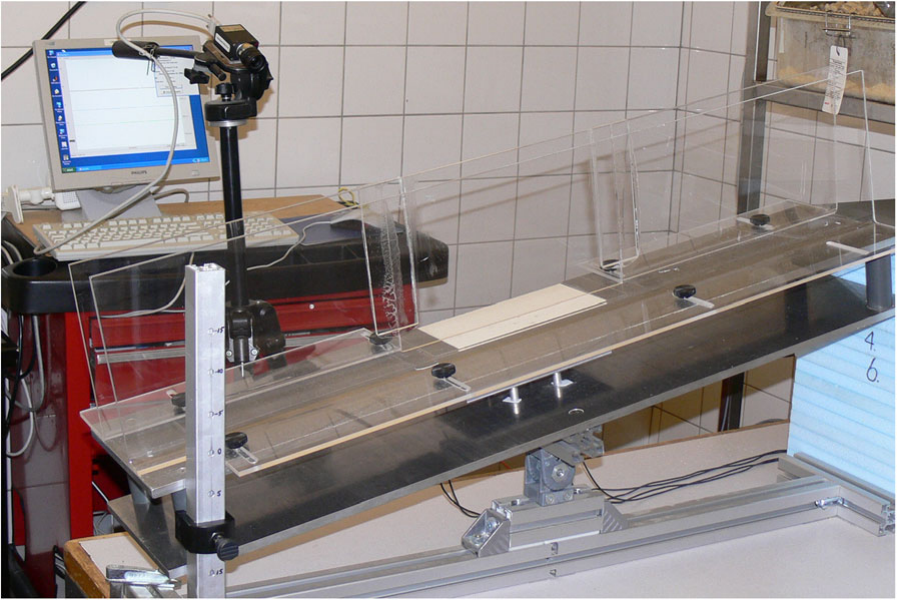
photograph of the custom made test set-up. It shows the Plexiglass walking tunnel, which had an adjustable slope and width. The KAPAplast force plate inlay (Fujifilm Sericol Nederland BV, Lochem, the Netherlands) that was supported by two horizontally and two vertically oriented 4.5 N load cells (L2357 S-Beam junior load cells, Futek, Irvine, USA) were placed in the bottom of the walking tunnel, which were connected to a computer with Matlab software (The Math Works Inc., Natick, MI). A camera recording the walking rats was also connected to the computer

**Fig. 3:**
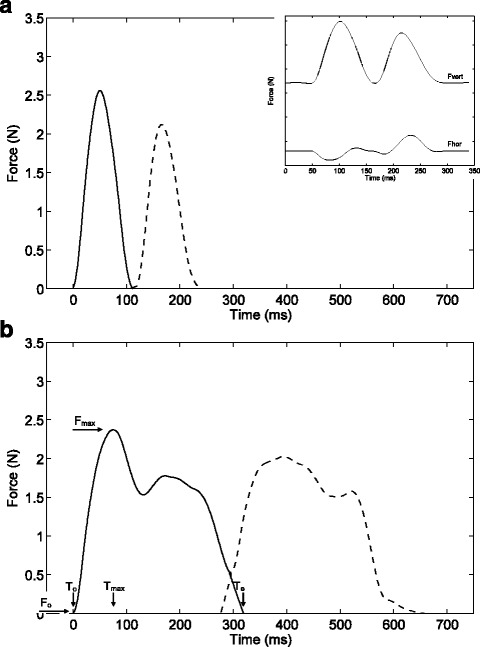
Example of force time series for SLOPE = 0°, LOAD = 0 (**a**) and SLOPE = 0°, LOAD = 1 (**b**) from the same rat. Forces represent resultant forces from the Fhor and Fvert vectors. First peak (closed line) represents FL, second peak (dotted line) represents HL. Insert shows separated horizontal (Fhor) and vertical force (Fvert) which corresponds with 1a. T_o_ = time at onset of peak; T_max_ = time at maximum force; T_e_ = time at end of peak; F_o_ = force at onset of peak; F_max_ = maximum force

The following variables were examined: stance time (limb to floor contact time, Tstance = T_e_-T_o_, Fig. 2b), peak force (Fmax, maximum force obtained during stance), total force measured during stance (Fint, impulse), and rate of force rise (RC, rising slope regression coefficient, Fig. 3b). If a measurement contained two contact points for a single limb, the mean values were used. Results are expressed as ‘least squares means’ and ‘standard errors of least squares means’, unless stated differently.

## Results

The training period did not affect body mass at the time of the measurements. Trained rats weighed 279±26 g, whereas non-trained rats weighed 280±20 g. Average additional load at 23 weeks was 64 ± 3.8 g.

Additional load visibly changed temporal as well as spatial characteristics of the gait. With additional load, the rats walked more slowly with larger stride width and reduced step size compared to without additional load (Fig. 5). Furthermore, with additional load the rat walked with a concave back.

**Fig. 4:**
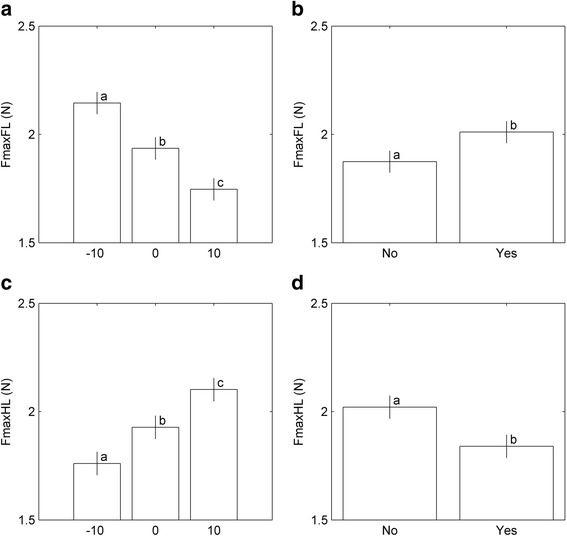
Effect of SLOPE (corrected for LOAD) on FL (**a**) and HL (**c**). Effect of LOAD (corrected for SLOPE) on FL (**b**) and HL (**d**) on Peak force (Fmax, the maximum force obtained during stance). Bars with different letters differ significantly (*P* < 0.0001). All bars represent the least square means estimates (± sem)

Table 1 shows load not significantly interacting with slope. Fmax was significantly affected by SLOPE (*p* < 0.0001)) as well as LOAD (all *p* < 0.0001) for both limbs: after adjustment for LOAD, an increase in SLOPE was associated to a decrease of FL Fmax and an increase of HL Fmax (Fig. 4a–c). After adjustment for SLOPE, LOAD resulted in an increase of FL Fmax (*p* < 0.0001) and a decrease of HL Fmax (*p* < 0.0001; Fig. 4b, d). See ﻿also ﻿Additional file 1: Table S1.

**Table 1:** Effect of SLOPE and LOAD on the examined variables: *P* values

Variable ^a^	RAT	SLOPE*LOAD ^b^	SLOPE	LOAD
FmaxFL (N)	0.0012	NS	<0.0001	<0.0001
FmaxHL (N)	0.0011	NS	<0.0001	<0.0001
DFmax (N)	0.0016	NS	<0.0001	<0.0001
TstanceFL (ms)	0.0015	0.0006	NS	<0.0001
TstanceHL (ms)	0.0014	<0.0001	0.0217	<0.0001
DTstance (ms)	0.0029	NS	0.0010	0.0004
FintFL (N.ms)	0.0013	<0.0001	<0.0001	<0.0001
FintHL (N.ms)	0.0012	0.0041	<0.0001	<0.0001
DFint (N.ms)	0.0019	0.0002	<0.0001	<0.0001
RCFL (N/s)	0.0013	<0.0001	<0.0001	<0.0001
RCHL (N/s)	0.0013	NS	0.0020	<0.0001
DRC (N/s)	0.0014	<0.0001	<0.0001	<0.0001

^a^ *FL* fore-limb, *HL* hind-limb, *D* FL-HL, *Fmax* peak ground reaction force, *Tstance* time that a paw has contact with the measuring plate, *Fint* total force measured during stance, impulse, *RC* rate of force rise

^b^NS, *P* > 0.01 In this case, the interaction SLOPE*LOAD was not included in

**Fig. 5:**
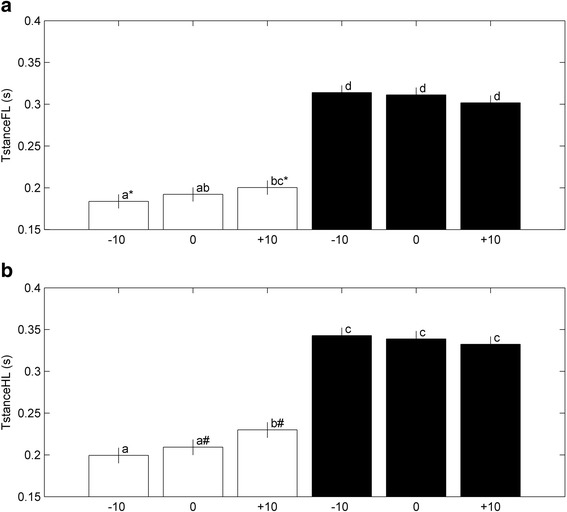
Effect of combinations of SLOPE and LOAD (white bars represent LOAD = 0, black bars represent LOAD = 1) on Stance time of FL (**a**) and HL (**b**). Bars with different letters differ significantly, *P* < 0.0001; bars with similar signs differ significantly (*, *P* < 0.01 and #, *P* = 0.0002). All bars represent least square means estimates (± sem)

Stance time was larger for both limbs with additional load compared to without additional load (Fig. 5). A slight, but significant difference of stance time (*p* < 0.01) was observed for both limbs between walking downhill and walking uphill without additional load. Stance time in the FL increased from 1.8 ± 0.7 s to 2.0 ± 0.8 s for −10 and +10, and stance time in the HL increased from 2.0 ± 0.9 s to 2.3 ± 0.9 s. However, SLOPE did not affect stance time when rats walked with additional load (Fig. 5). A significant interaction between LOAD and SLOPE was found for stance time in FL (*p* = 0.0006) and HL (*p* < 0.0001).

**Fig. 6:**
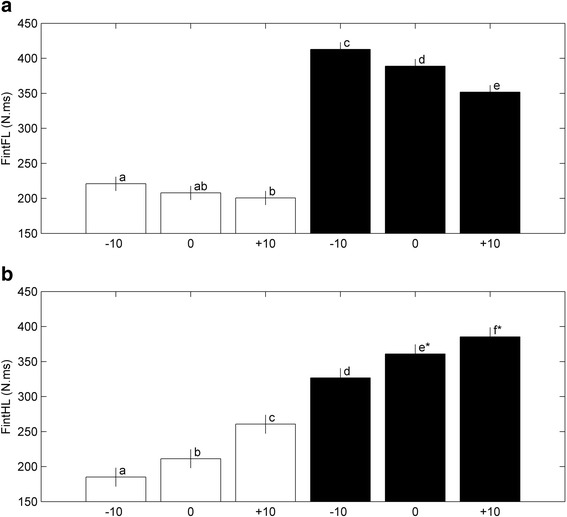
Effect of combinations of SLOPE and LOAD (white bars represent LOAD = 0, black bars represent LOAD = 1) on impulse (Fint) of FL (**a**) and HL (**b**). Bars with different letters differ significantly, *P* < 0.0001. Bars with similar sign (*) differ significantly, *P* = 0.0007. All bars represent the least square means estimates (± sem)

For both limbs, Fint was largest with additional load (*p* < 0.0001; Fig. 6). For FLs, an increase in SLOPE was associated with a decrease of Fint that was larger with additional LOAD than without (Fig. 6a). SLOPE was associated with Fint with additional load and without, only for the hind limb (Fig. 6b).

**Fig. 7:**
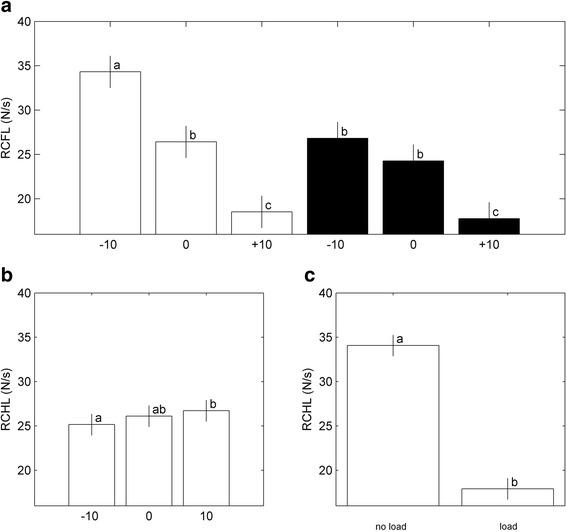
Effect of combinations of SLOPE and LOAD on Rate of force rise of FL (FLRC) (**a**). Effect of SLOPE (after correction for LOAD) on HLRC (**b**). Effect of LOAD (after correction for SLOPE) on HLRC (**c**). White bars represent LOAD = 0, black bars represent LOAD = 1. Bars with different letters differ significantly, *P* < 0.001, bars with similar sign (*) differ significantly, *P* = 0.0001. All bars represent the least square means estimates (± sem)

Hind-limb rate of force rise (HLRC) was significantly affected by SLOPE (*p* < 0.0001) as well as LOAD (*p* < 0.0001; Table 1). An increase in SLOPE resulted in a significantly lower fore-limb rate of force rise (FLRC) without LOAD, and a significantly lower FLRC for +10° compared to slopes −10° and 0° with LOAD (Fig. 7a). The largest FLRC was found for LOAD = 0, SLOPE = −10°. Though significant, HLRC was less affected by SLOPE (*p* = 0.002; Fig. 7b). LOAD, however, resulted in a large decrease of HLRC after correction for slope (Fig. 7c).

**Fig. 8:**
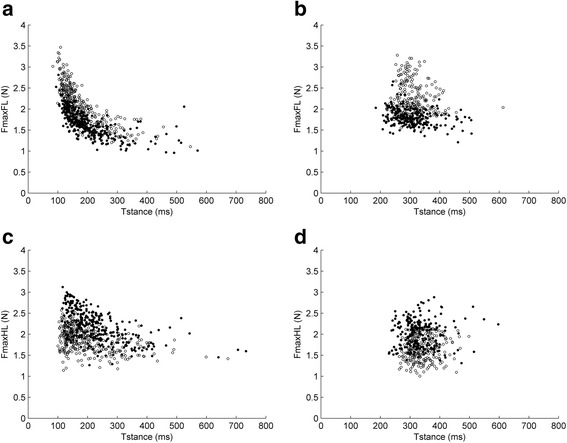
Relation of Fmax to stance time for FLs (**a**, **b**) and HLs (**c**, **d**), for LOAD = 0 (**a**, **c**) or LOAD = 1 (**b**, **d**). Each dot represents a measurement. White dots represent SLOPE = −10°, black dots represent SLOPE = +10°

Figure 8 presents the association of Fmax to stance time for FLs and HLs for SLOPE = −10° and SLOPE = +10°. The associations remained stable for the two slopes. For LOAD = 0, FL Fmax decreased parabolically, while stance time increased (Fig. 8a). This association was less pronounced for HL Fmax (Fig. 8c). For LOAD = 1, Fmax and stance time in both limbs were not associated (Fig. 8b, d).

### Discussion

This study aimed to test the effects of additional load and slope on ground reaction forces in rat FL and HL. In summary, in female adult Wistar rats, the FL bones showed the highest Fmax when the rat walked downward, while the HL bones showed the highest Fmax when the rat walked upward. Additional load increased peak force in the FLs but decreased peak force in the HLs. Moreover, additional load increased stance time. Overall, this suggests that upward walking without load generates a response in the HL, while downward walking generates a response in the FL. The additional load likely leads to changes in the FL bones when walking downward, though bone changes in the HL may remain undetectable.

The results revealed that walking downward without load increased FL peak force as well as rate of force rise, while only walking upward without load increased HL peak force. HL rate of force rise remained stable for any exercise. The effectiveness of the high impact exercise on bone is therefore expected to be higher for the FL than for the HL. Yet, exercises with the highest impact induced comparable peak forces and rates of force rise for FL and HL. Skerry (1997) suggests that bone adaptation is controlled especially by loads that cause a change in habitual strain magnitude. Since, in the present study, the rats were housed in horizontal level cages, it is most likely that habitual loading consists of ground reaction forces at the zero-degrees slope without load.

The results similarly showed that additional load significantly increased stance time of FL and HL, which indicates concurrent walking velocity decreased. Furthermore, additional load affected the relation of peak force to stance time, which suggests that the different rats may have used different strategies to cope with the load, resulting in a less predictable peak force and rate of force rise in relation to stance time. The relations of peak force to stance time also revealed that high peak force (comparable to that when walking with additional load) and high rate of force rise (comparable to that when walking without additional load) in FL can be obtained by increasing the walking velocity with a motor driven treadmill.

The results similarly show that independent of slope, the load reduced HL peak force. Since ground reaction force of the HL is larger when walking with a load for all slopes, the pattern of the ground reaction force may change with a longer stance time. In line with the results, Gillis and Biewener (2002) reported that rat HL muscle activity of the biceps femoris and vastus lateralis increased with an increase in slope from −15° to +15°. Other studies that reported peak ground reaction forces of rat FL and HL were restricted to separate vertical and/or caudio-cranial and/or medio-lateral peak force or to vertical impulse during level walking (Clarke et al. 1997; Webb and Muir 2004). For female Wistar rats, Clarke (1995) and Webb and Muir (2003) confirmed that vertical force data reflected the results of peak ground reaction force as well as impulse of FL in relation to HL walking at level.

A potential limitation of this study was the fact that blind investigations could not be performed, because the investigator needed to be able to detect the load in the backpack and see the declining or inclining slope. In addition the investigator needed to screen all video recordings to check the usefulness of the measurements. However, all force plate measurements were recorded automatically, which means the investigator itself had no influence on the results. Another limitation is that the present study assumed comparability of contralateral limbs. Moreover, mediolateral components of ground reaction force were assumed to te negligible (Muir and Whishaw 1999) and were therefore not included in the measurement.

A strenghth of the study is that a non-invasive physiological training method was used to create a sufficient stimulus for mechano-response of bone, which could also have an application in people. However extrapolation towards people is restricted, since people are bipeds while rats are quadrupeds. Nevertheless, extrapolation from rodents towards people is common in research on several diseases. For instance, in osteoarthritis, the gait abnormalities seen in rodent models and in humans reflect similar compensatory behaviors (Jacobs et al. 2014). Only in animals that are both bipedal and quadrupedal, the two types of locomotion can be compared. In chimpanzees, Pontzer et al. compared bipedal locomotion with quadrupedal locomotion, which resulted in similar spatiotemporal characteristics (Pontzer et al. 2014).

## Conclusions

In conclusion, in female adult Wistar rats, the FL bones show the highest response to mechanical loading when the rat walks downward, while the HL bones show the highest response when the rat walks upward. Since additional load could change posture in addition to temporal and spatial characteristics of gait, exercises without load may be preferable in these circumstances. Peak force and rate of force rise change in comparison to the habitual exercise, which suggest that downward exercise should be the preferred exercise to study FL mechano-sensitivity. These data on ground reaction forces during walking are important for the design of future animal studies that aim to test mechanoresponse in a non-invasive physiologic training method.

## Additional file

Additional file 1: Table S1. Effect of SLOPE and LOAD on the Least square means of measured variables. (DOCX 18 kb)

## Abbreviations

Fint: Impulse, total force measured during stance
FL: Fore-limb
Fmax: Peak force, maximum force obtained during stance
HL: Hind-limb
RC: Rate of force rise, rising slope regression coefficient
Tstance: Stance time, limb to floor contact time
