# Supplementary material for: Ground reaction forces during walking with different load and slope combinations in rats
Source: J Exp Orthop. 2017 Aug 31;4:28. doi: 10.1186/s40634-017-0102-8 (PMC5578952; doi:10.1186/s40634-017-0102-8)
Supplement: Additional file 1: Table S1. — Effect of SLOPE and LOAD on the Least square means of measured variables. (DOCX 18 kb) [file 40634_2017_102_MOESM1_ESM.docx]

Additional file 1: Table S1. Effect of SLOPE and LOAD on the Least square means of measured variables.

| Additional weight | no |  |  | no |  |  | no |  |  | yes |  |  | yes |  |  | yes |  |  |
| --- | --- | --- | --- | --- | --- | --- | --- | --- | --- | --- | --- | --- | --- | --- | --- | --- | --- | --- |
| SLOPE | -10 |  |  | 0 |  |  | +10 |  |  | -10 |  |  | 0 |  |  | +10 |  |  |
| n | 329 |  |  | 341 |  |  | 333 |  |  | 248 |  |  | 232 |  |  | 238 |  |  |
| Fmax FL (N) | 2,11 | ± | 0,46 | 1,89 | ± | 0,44 | 1,68 | ± | 0,32 | 2,23 | ± | 0,39 | 2,01 | ± | 0,3 | 1,81 | ± | 0,21 |
| Tstance FL (ms) | 180 | ± | 70 | 190 | ± | 90 | 200 | ± | 80 | 310 | ± | 50 | 320 | ± | 60 | 310 | ± | 60 |
| Fint FL (Ns) | 218 | ± | 64 | 212 | ± | 67 | 199 | ± | 64 | 409 | ± | 78 | 394 | ± | 69 | 355 | ± | 75 |
| RCFL (N/s) | 35 | ± | 17 | 27 | ± | 15 | 18 | ± | 9 | 28 | ± | 12 | 24 | ± | 13 | 17 | ± | 11 |
| Fmax HL (N) | 1,86 | ± | 0,28 | 2,05 | ± | 0,34 | 2,19 | ± | 0,36 | 1,67 | ± | 0,3 | 1,85 | ± | 0,29 | 2 | ± | 0,32 |
| Tstance HL (ms) | 200 | ± | 90 | 210 | ± | 70 | 230 | ± | 90 | 340 | ± | 40 | 340 | ± | 50 | 330 | ± | 60 |
| Fint HL(Ns) | 182 | ± | 82 | 216 | ± | 73 | 256 | ± | 93 | 322 | ± | 74 | 367 | ± | 90 | 387 | ± | 96 |
| RCHL (N/s) | 34 | ± | 10 | 35 | ± | 11 | 35 | ± | 12 | 16 | ± | 5 | 18 | ± | 6 | 19 | ± | 7 |

FL = fore-limb; HL = hind-limb; Fmax = peak ground reaction force; Tstance = time that a paw has contact with the measuring plate; Fint = total force measured during stance, impulse; RC = rate of force rise.
